# Selection and Outcomes in Abdominoperineal Resection

**DOI:** 10.3389/fonc.2020.01339

**Published:** 2020-08-18

**Authors:** Norbert Garcia-Henriquez, Daniel J. Galante, John R. T. Monson

**Affiliations:** Advent Health Medical Group, Center for Colon and Rectal Surgery, Orlando, FL, United States

**Keywords:** indication, selection, outcomes, intersphincteric APR, extrasphincteric APR, extralevator abdominoeperineal excision (ELAPE)

## Abstract

Since the initial descriptions of the abdominoperineal resection by Sir William Ernest Miles which was then followed by the perfection of the total mesorectal excision by Professor Bill Heald, the surgical management of rectal cancer has made tremendous strides. However, even with the advent and sophistication of neoadjuvant therapy, there remains a formidable amount of patients requiring an abdominoperineal resection. The purpose of this review is to delineate the indication and selection process by which patients are determined to require an abdominoperineal resection, as well as the oncologic and overall outcomes associated with the operation.

## Introduction

Historically, rectal cancers have a mortality of 100% if left untreated. The eighteenth and nineteenth century contributions of Morgagni, Lisfranc, Kocher, Kraske, Gaussenbauer, and Czerny all helped create a better understanding of the surgical management of rectal cancer (1, 2). Despite not being the first to attempt rectal resection from both an abdominal and perineal approach, until standardized by Sir William Ernest Miles, the surgical procedure for the management of rectal cancer was the perineal proctectomy. These patients ultimately suffered a nearly 90% recurrence rate and an incredibly high mortality rate. In 1908 Dr. Miles first published his early series of 12 patients undergoing abdominoperineal resection (APR). Following his well-documented description of the procedure and principles of technique, the APR became the standard of care for surgical management of rectal cancer. His technique followed a stepwise approach. This included creation of a colostomy (abdominal anus), removal of the “pelvic colon” with its blood supply to interrupt the zone of upward spread of disease, resection of the pelvic mesocolon, iliac lymphadenectomy, and extralevator perineal dissection (3, 4).

Over the years there have been additional improvements and adjustments to the APR technique (and the treatment of rectal cancer). The synchronous approach, introduced in the 1930s, allowed two surgical teams to perform the abdominal and perineal dissections simultaneously (5–7). Studies also showed that most lymphatic spread is cephalad, ultimately allowing the distal rectum to be spared at the time of resection, and thus laying the groundwork for the low anterior resection (3, 8–10). Data on surgical margins has only strengthened the argument for anterior resection and preservation of the sphincters; the original accepted distal margin of 5 cm has reduced to as little as 1 cm or less (11). Of late, some have postulated that a gross negative margin is oncologically sound. With time, the application of the abdominoperineal resection has decreased.

While Dr. Miles' description of proctectomy included the principles of total mesorectal excision (TME), it was not until the 1980's when Professor Heald's initial description of the TME that put this facet of the procedure into the forefront of rectal cancer surgery. TME involves removal of the rectum and associated lymphoid tissue as a single unit within the encasement known as the mesorectal fascia. This envelope separates the rectum and mesorectum from the surrounding structures and follows a “holy plane” into the pelvis (12–14). Today it is the standard dissection technique in rectal cancer surgery.

With improvements in surgical technique and instrumentation (e.g., surgical stapler, advanced electrocautery devices, etc.), as well as the introduction of neoadjuvant treatments, the surgical treatment of rectal cancer has advanced and matured. No longer are all patients with operable rectal cancers faced with the prospect of a permanent colostomy and a potentially morbid perineal wound. Anterior resection has proven to be a successful operation for the treatment of most rectal cancers, even those that are very low. And while the concept of TME has emphasized sphincter sparing surgery, the fact remains that local recurrence due to incomplete TME or violation of the principles of total mesorectal excision must remain in the front of every rectal surgeon's mind, regardless of the wish for sphincter preservation (15–17).

## Indications For Abdominoperineal Resection

Unfortunately, despite the major advancements in the multidisciplinary approach to rectal cancer, approximately 40% of patients with rectal cancer will undergo an abdominoperineal resection, resulting in a permanent colostomy.

The utility of the abdominoperineal resection lies in the ability to remove the low (defined by tumors within 5 cm from the anal verge) tumor, associated lymphoid tissue, and involved structures from within the deep pelvis. Indications for APR include ultra-low rectal tumors with inability to obtain a negative distal margin, involvement of the external sphincter or invasion of the levator ani complex. Those patients with poor baseline sphincter function with rectal cancer are also well-suited for abdominoperineal resection. While much of the discussion over indications for APR focuses on rectal cancer, it should not be overlooked that the APR is the salvage procedure of choice for anal squamous cell carcinoma, as well as those patients with rectal dysplasia in the setting of inflammatory bowel disease, not amenable to gastrointestinal continuity restoration. For simplicity and clarity, the discussion of workup and indications for APR will focus on patients with rectal cancers.

## Patient Evaluation and Work-up

In evaluating a patient with rectal cancer, the standard principles as dictated by the American Society of Colon and Rectal Surgeons and other professional surgical societies should be followed. Obtaining a thorough history and physical examination are the critical initial steps that are to be undertaken. This should be followed by a formal endoscopic evaluation, as well as complete staging with CT scans of the chest, abdomen and pelvis. For local staging, an MRI should be obtained utilizing a dedicated “Rectal Cancer Staging” protocol. The MRI is key in helping to determine local invasion, recognition of mesorectal (and extra-mesorectal) lymph nodes, circumferential resection margin, and sphincter involvement. The endorectal ultrasound is no longer considered standard in the workup of rectal cancer and is reserved for those patients unable to obtain an MRI or in some T1 or T2 lesions. A baseline carcinoembryonic antigen (CEA) level should be obtained and then obtained based on the National Comprehensive Cancer Network guidelines during the surveillance period. Additionally, all patients with rectal cancer should be presented and discussed at a multidisciplinary conference (MDT) with surgeons, medical and radiation oncologists, pathologists, and radiologists. This has become yet another standard step in the management of rectal cancer. Once the plan of care has been established by multidisciplinary consensus, the patient should be connected to a dedicated nurse navigator (or other similar individual) to help shepherd the patient through the treatment process (18, 19).

As the APR is a large, invasive procedure; it goes without saying that this procedure is not performed without extensive patient counseling and planning. After the completion of neoadjuvant treatment (either chemoradiotherapy or total neoadjuvant treatment), the patient should be appropriately re-staged via a triple evaluation. This involves a complete physical examination, repeat endoscopic evaluation with biopsies, and complete radiographic evaluation with CT and MRI imaging. Thereafter, the patient is once again presented at the MDT conference prior to surgical intervention.

Upon evaluation of the re-staged patient, those patients with persistent tumors on physical or endoscopic exam, radiographic evidence of residual tumor with sphincter or levator involvement or suspicious low mesorectal lymph nodes should be referred on for APR. As these patients will have undoubtably received radiation therapy as part of their neoadjuvant treatment, discussion of perineal wound management with possible flap closure (vertical rectus abdominus- VRAM, gracilis muscle flap) should result in the associated plastic surgery consultation (20). Patients are also referred to a certified wound and ostomy care nurse (WOCN) for stoma marking and education (21).

Ultimately it is the job of the operating surgeon to counsel the patient as to the risks and benefits of the proposed surgical procedure. While not always discussed in the consultation room, sexual function, urinary function, body image, and other quality of life indicators should all be thoroughly reviewed (22–24).

## Operative Technique

**Figure 1:**
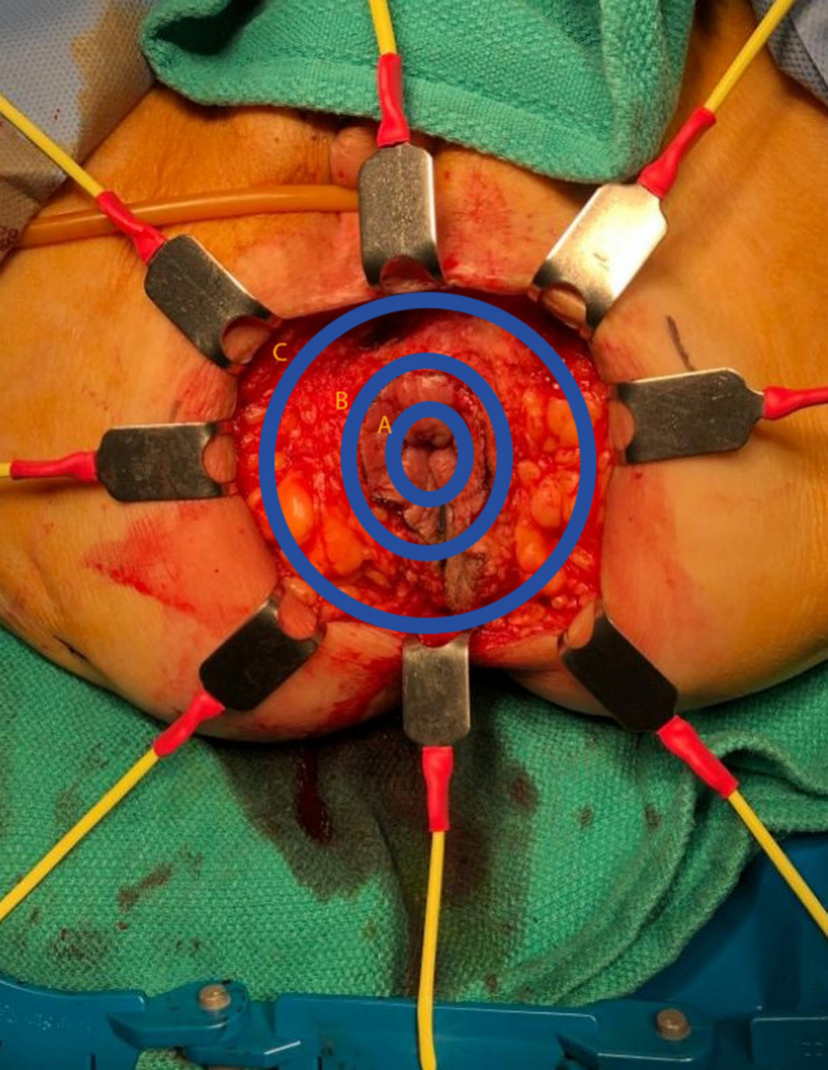
Plane of perineal dissection for APR. **(A)** Intersphincteric dissection; **(B)** Extrasphincteric dissection (standard APR); **(C)** Extralevator abdominoperineal excision (ELAPE).

Once determined that an abdominoperineal resection is required, the next step is to decide which technique (intersphincteric Figure 1A, extrasphincteric Figure 1B, or extralevator Figure 1C) will be used and what approach (open, laparoscopic, or robotic) will best suit the patient. Specifically, when deciding on which technique will be performed, it is of critical importance to carefully review the MRI at the time of the initial diagnosis as well as following neoadjuvant therapy with specific focus on the relationship of the tumor to the mesorectal fascial envelope; the circumferential radial margin (CRM). This should all be done at the institutional multidisciplinary evaluation. Regardless of what technique is undertaken, the goals of an abdominoperineal resection include extirpation of the distal colon, rectum, mesorectum, and anus to negative margins followed by the creation of the end colostomy.

The operation is divided into two phases: the abdominal phase and the perineal phase. Whether this is performed sequentially or simultaneously is at the discretion of the operating surgeon. The same could be said regarding performing the perineal portion in the lithotomy or prone position.

The abdominal phase, regardless of the approach undertaken, is commenced by a formal intra-abdominal survey to assure the absence of metastatic disease. This is generally followed by mobilization of the distal sigmoid colon from either the medial or lateral approach, identifying the critical retroperitoneal structures, isolating and dividing the inferior mesenteric artery (IMA) and entering the total mesorectal excision (TME) plane. During this step of the operation, care should be taken to identify the superior hypogastric (sympathetic) nerves which are intimately associated with the aortic bifurcation and the origin of the IMA as one enters the pre-sacral space. Once identified and kept away from the plane of dissection, the dissection is then carried in a posterior to lateral to anterior trajectory with the goal of reaching the pelvic floor at the level of the levator ani complex. Along this dissection, the surgeon should remain cognizant of remaining within the avascular plane as violation of this plane may result in injury to the presacral venous plexus which can be associated with catastrophic hemorrhage. Next, in a cylindrical fashion the lateral to anterior portion of the dissection is commenced at the level of the peritoneal reflection. Once encountered, the lateral stalks are divided. Upon approaching the anterolateral area of the dissection, again the surgeon should be cognizant of the inferior hypogastric (parasympathetic) nerves which is intimately associated with the seminal vesicles and prostate gland in males and the vagina in females. Once the rectum is circumferentially mobilized to the level of the levator ani complex, the colon is then divided proximally, and the end colostomy is created. In our institution, we routinely perform the perineal portion in the prone position and customarily, once the abdomen is closed and the colostomy matured, the patient is turned to the prone position with the assistance of the operating room staff, the surgeon and the anesthesia staff.

In preparation for the perineal phase, the gluteal area is separated with tapes for proper perineal exposure. Once the patient is properly prepped and draped, the incision is marked, laterally at the ischiorectal fossae, the perineal body anteriorly and the coccyx posteriorly. A purse string suture is used to close the anus. An elliptical incision is made encompassing the marked sites and a circumferential dissection is undertaken. In the traditional (extrasphincteric) APR, the dissection plane is just outside the external sphincter muscle and carried out cephalad to and through levator ani complex, meeting the intra-abdominal dissection. There are several aspects of this dissection that are of importance. First, the target landmark while in the posterior dissection plane is the coccyx. Of note, the coccyx may be resected in effort to obtain better visualization or in *en bloc* fashion to obtain an appropriate margin. It should be reiterated that the posterior dissection is to remain within the avascular plane to avoid inadvertent vascular injuries to either the presacral venous plexus or the internal iliac vessels, which can be quite challenging to control and may result in catastrophic hemorrhage. It is within this plane that the abdominal cavity is accessed by dividing the anococcygeal ligament. Secondly, laterally the dissection is carried out just outside the external sphincter within the ischiorectal fossae in a cephalad direction, going through the levator muscle and entering the abdominal cavity. Once the latter two portions are completed, attention is then focused to the anterior dissection plane. This part of the operation should invoke pause to the surgeon. As previously described, there are several critical structures that need to be accounted for in this area, specifically the vagina in females and seminal vesicles/prostate in males, in addition to the urethra and trigone muscle of the urinary bladder, an injury which is associated with a high level of morbidity. Once the rectum is completely mobilized circumferentially, it is extracted through the perineal wound. It is our practice to evaluate, grade and photograph the specimen prior to sending for pathologic evaluation. Lastly, the perineal wound is closed in layers and a Jackson-Pratt or Blake drain is left in the pelvis at the discretion of the operation surgeon.

Although the intersphincteric APR is mostly used in those patients with inflammatory bowel disease, specifically Crohn's disease, it is also indicated and reserved for those patients with an ultralow rectal cancer in which the sphincter complex is spared yet requiring a distal transection to secure an appropriate distal resection margin. From a technical perspective, it is quite like the traditional APR, with the exception that the dissection is carried out within the intersphincteric space with the goal of removing the mucosa, submucosa, and muscularis propria of the rectal wall, leaving the external sphincter intact. Leaving the external sphincter *in situ* offers the advantage of easier re-approximation at the time of perineal closure.

**Figure 2:**
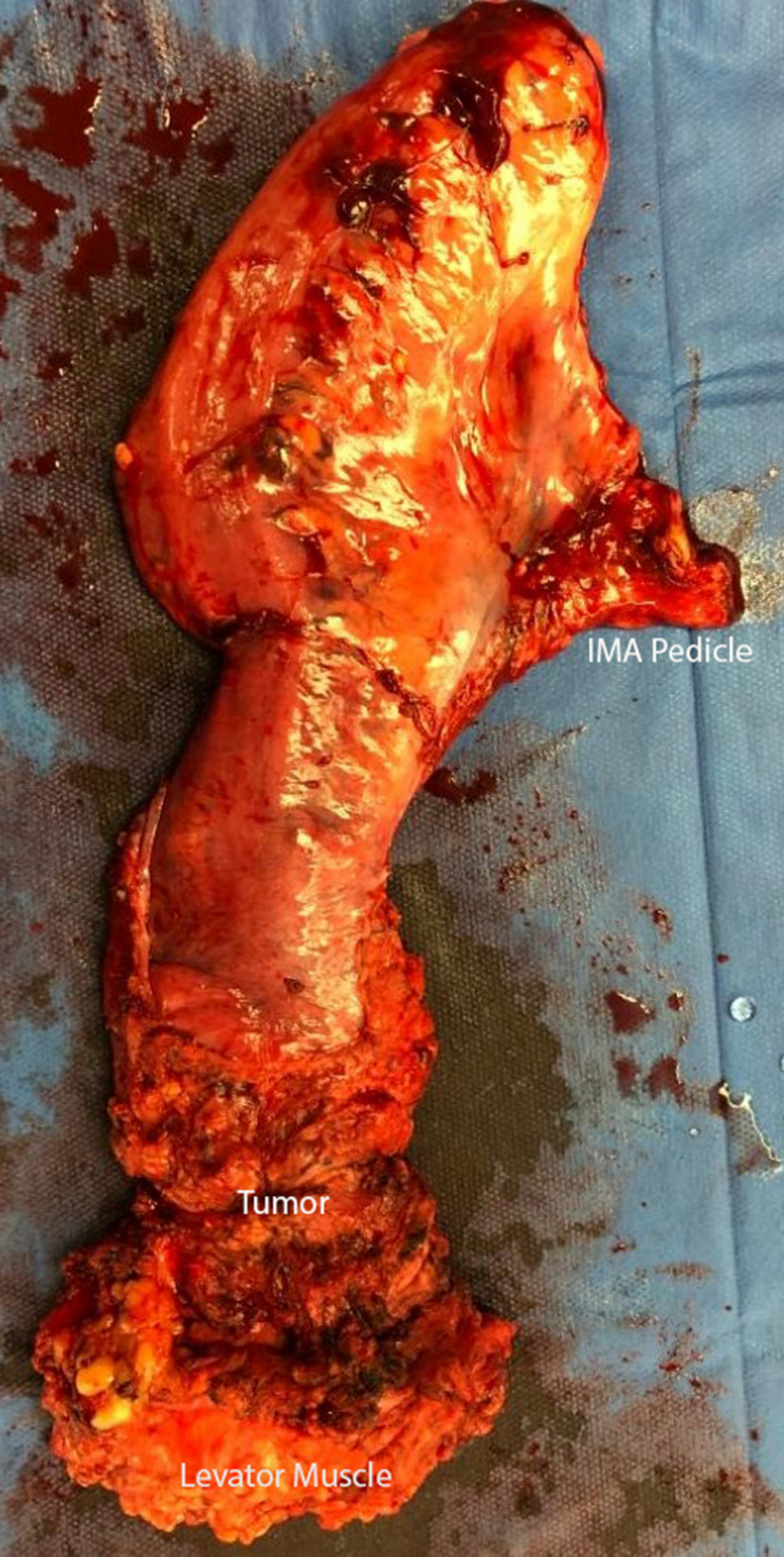
Complete ELAPE specimen.

**Figure 3:**
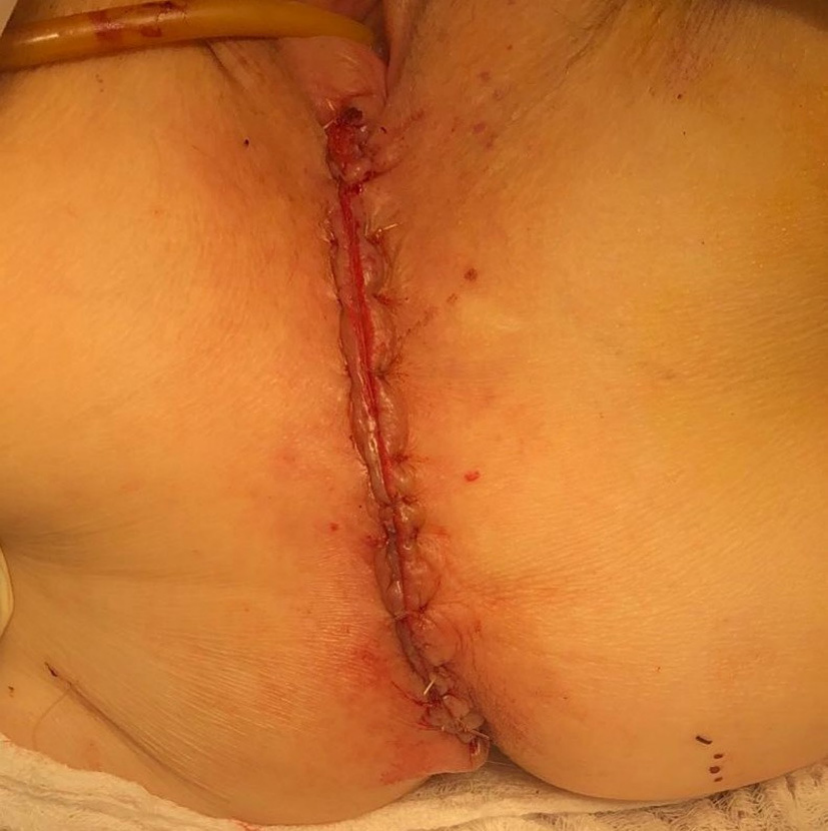
Perineal wound closure following ELAPE.

The extralevator abdominoperineal excision (ELAPE) is indicated specifically for low rectal tumors involving the levator complex. In this approach, the pelvic dissection in the abdominal phase typically ends several centimeters above the levator complex followed by standard colostomy creation and abdominal wall closure whereas the perineal phase differs in that the lateral dissection is to the level where the levator complex inserts into the pelvic sidewall to remove more tissue and avoid perforation of the specimen Figures 1–3. This wider lateral dissection affords the ability to obtain an R0 resection and a negative circumferential resection margin, while potentially creating a larger, more difficult to close, perineal defect.

## Outcomes

The benefits of minimally invasive surgery are well-established. When it comes to rectal cancer surgery, the benefits of the minimally invasive approach when compared to the traditional open approach, are in line with the status quo of improved cosmesis, decreased lengths of stay, and earlier return of bowel function. However, in recent years many have questioned if the minimally invasive approach affords patients similar results from an oncologic perspective. Fleshman et al., in the initial Z6051 trial where 23.3% of the patient cohort underwent an abdominoperineal resection found that laparoscopic resection compared with open resection failed to meet non-inferiority criteria for pathologic outcomes, specifically when evaluating the circumferential radial and distal margins (25).

Interestingly, in a recent follow up to this trial, the same group concluded that laparoscopic resection was not significantly different to open resection based on the outcomes of disease-free survival, local and distant failures (26). Similarly, the ALaCaRT trial by Stevenson et al., where 10% of the study cohort underwent abdominoperineal resection, could not establish non-inferiority when comparing laparoscopic and open resection for rectal cancer (27).

Although there have been tremendous strides made in the management of rectal cancer specifically since the introduction of the total mesorectal excision and neoadjuvant chemoradiation therapy, local failures continue to be a significant challenge in these patients. This is especially relevant in those patients undergoing an abdominoperineal resection where the local failure rate is thought to be on the order of approximately 10%. In general, compared to those patients who undergo a low anterior resection, those subjected to an abdominoperineal resection have worse outcomes, specifically, higher local failure rates, higher CRM positivity, and decreased overall survival (2).

Despite the introduction of the total mesorectal excision by Dr. Heald over 30 years ago, the frequency of CRM positivity has not changed as one might expect. It is hypothesized by many that there is a higher incidence of specimen perforation in patients with low rectal tumors. Anatomically, the lower rectum is different than the upper and mid rectum in the sense that the mesorectum is absent at this level thus dissection is often within the muscularis propria layer, resulting in a “coned in” appearing specimen which results in a positive CRM in approximately 20% specifically those undergoing an abdominoperineal resection. Given this phenomenon, the idea of performing a wider dissection, encompassing the entire levator complex came about. In 2010, Holm's group studied the influence of the operating position on outcomes. They compared 176 extralevator abdominoperineal excisions (ELAPE) with 124 standard abdominoperineal resection procedures performed in 11 European centers. In the ELAPE group, 127 procedures were performed with the patient in the prone position and 30 were performed with the patient in the lithotomy position (the position was not specified in 19 cases). For ELAPE, the tumor perforation rate was significantly lower in the prone group than in the lithotomy group (6.4% vs. 20.6%, *p* = 0.027). The two groups did not differ significantly in terms of the TNM grade, the aim of the operation, tumor size, tumor depth, circumferential resection margin status, sex, age, and preoperative chemotherapy status. The main limitation was that preoperative radiotherapy was applied more frequently in the prone group than the lithotomy group (101/122 vs. 18/30; *p* = 0.012). The researchers did not comment on the duration of the patients' radiotherapy or why one position was chosen over the other. None the less it should be noted that, even in a multivariate analysis, the prone position was an independent factor for protection against tumor perforation (odds ratio, 0.12; 95% confidence interval, 0.02–0.67; *p* = 0.016) (28).

Similarly, Han et al., in a randomized clinical trial of conventional vs. cylindrical abdominoperineal resection found lower rates of CRM involvement and specimen perforation in favor of the cylindrical technique (29). Contrarily, Prytz et al. studied 519 patients undergoing extralevator abdominoperineal resection and found that compared to conventional APR, the former had a higher local failure rate at 3 year follow up, however in those patients with tumors within 4 cm from the anal verge, local failure rates were not statistically significant. Additionally, no overall survival difference was found in between the two groups (30). Overall, the results of the data on long-term oncologic outcomes are mixed. There are some studies, however that favor the extralevator abdominoperineal resection, especially in those patients with T3 and T4 tumors (31).

Of recent there have been multiple meta analyses, cohort studies, and randomized control trials to analyze the differences between the standard APR and the ELAPR. A randomized control trial from a multicenter, international group examined the outcomes of 34 patients (17 conventional APR, 17 ELAPR) with an endpoint of circumferential resection margin. They noted significantly better circumferential margin status in the ELAPR group, with no difference in intraoperative tumor perforation or other complications (32). Additionally, Carpelan et al. compared their APR cohort (27 patients, years 2004–2009) with ELAPR patients (42 patients, years 2009–2016) and noted no significant differences between the groups (33). There was, however a trend toward improved circumferential margin, decreased intraoperative perforation and lower rates of local recurrence in the ELAPR cohort. Additional studies and their findings are listed in Table 1.

**Table 1:** Studies comparing standard abdominoperineal resection (APR) and extralevator abdominoperineal excision (ELAPE).

**Study**	**Year**	**Style of Study**	**Included Studies (No. Patients)**	**Outcomes**
Qi et al. (38)	2019	Meta-Analysis	17 studies (4,049 patients; 2,248 ELAPR, 1,801 APR)	N/S margins or perineal wound complications; Lower intraoperative perforation and lower recurrence
Lehtonen et al. (39)	2019	Cohort study	206 patients	No significant increase in survival with ELAPR, decrease in positive CRM with ELAPR, Lower perforation rate with ELAPR
Zhang et al. (40)	2017	Meta-Analysis	17 studies (3,479 patients; 1,915 ELAPR)	Significantly lower 3 year recurrence, local recurrence, mortality, perforation and improved circumferential margin with ELAPR
Bianco et al. (32)	2017	RCT	34 patients	Significantly better CRM with ELAPR; no difference in intraoperative perforation or other complications
Negoi et al. (41)	2016	Systematic Review	1 RCT, 10 non-randomized comparative studies (3,056 patients; 1,736 ELAPR, 1,320 APR)	ELAPR has lower intraoperative perforation rate, no significant differences in CRM, R0 resection, local recurrence. Lower blood loss in ELAPR
Güven and Aksel (42)	2019	Retrospective Review	104 patients	Significantly higher postop complications with ELAPR; Similar CRM, LN yield, intraoperative perforation and 2 year local recurrence
Carpelan et al. (33)	2018	Retrospective Review	69 patients; (27 APR, 42 ELAPR)	Trend toward significance for ELAPR in CRM, Intraoperative perforation and LR
De Nardi et al. (43)	2015	Systematic Review	1 RCT, 5 Case Control, 52 Cohort Studies	ELAPR has better CRM, decreased intraoperative perforation, lower local recurrence and lower LOS; Higher wound complication rates

Along the lines of outcomes, some have postulated that perhaps the position may influence overall oncologic outcomes. Although the data regarding the oncological success of the traditional lithotomy vs. prone positions are mixed, there is consensus that the prone position is a less cumbersome operation for the surgeon and associated with decreased blood loss and operative time, recent studies have failed to demonstrate a formidable difference with regards to intraoperative specimen perforation or circumferential radial margin positivity (34).

Given the nature of the operation, patients undergoing abdominoperineal resection are subjected to numerous post-operative complications including small bowel obstruction and surgical site infection, both superficial and deep. The most notable and associated with the highest level of morbidity is the perineal complication, specifically the perineal dehiscence and hernia. Most data would quote a perineal complication rate of 40–45%, however in the era of neoadjuvant chemotherapy, the rates can be as high as 60–70%. In techniques such as the intersphincteric and extrasphincteric resections, more tissue is spared, thus affording the surgeon the theoretical advantage of approximating the perineal wound with a bit more ease. Nonetheless, the advantage remains theoretical as the wound morbidity remains considerable. In the extralevator technique, because the dissection encompasses the lateral most edge of the ischiorectal fossae to the level of levator insertion at the pelvic side wall, a sizeable perineal defect remains with very little proper quality tissue available for re-approximation, posing quite the challenging task. Han et al., retrospectively evaluated 228 rectal cancer patients who underwent an extralevator abdominoperineal excision and subsequently compared those who had a perineal mesh repair to primary repair. Of note, 51% of these patients were treated with neoadjuvant radiation therapy. They concluded that overall perineal complications were significantly higher in those undergoing primary closure (15% vs. 35%; *p* = 0.001). Additionally, on multivariate analysis, they noted preoperative radiation therapy, conventional primary closure, and intraoperative specimen perforation were significantly associated with perineal procedure-related complications (35). Although the perineal dissection of the APR may be completed in the prone or lithotomy posisition, some who perform the operation with the patient prone are of the opinion that it is associated with less morbidity and better oncologic outcomes. De Campos-Lobato, in a 2011 retrospective study of 168 patients, demonstrated no difference in perioperative morbidity and oncologic outcomes and should be left at the surgeons' discretion (36).

To isolate the pelvic cavity and perineal wound from the remainder of the abdominal cavity, some have advocated for an omentoplasty following abdominoperineal resection. Blok et al. in a retrospective study of 106 patients who underwent an omentoplasty, demonstrated that omentoplasty did not lower the incidence or duration of perineal morbidity in patients undergoing abdominoperineal resection for rectal cancer. Additionally, they found that the omentoplasty itself was associated with a risk for reoperation (37).

## Conclusion

The management of rectal cancer continues to be quite challenging despite the tremendous strides that have been made into the modern era of colon and rectal surgery. Specifically, as advancement in surgical technique has evolved, there are still about 40% of patients that cannot undergo restoration of gastrointestinal tract continuity and unfortunately an abdominoperineal resection must be undertaken. Upon completion of the standard work up, it is of critical importance that all patients with rectal cancer be presented at the institutional multidisciplinary evaluation as this has been shown to positively influence patient outcomes. It must be reiterated that it is the commitment of the operating surgeon to discuss the risks and benefits of the operation with the patient in thorough detail, including sexual and urinary dysfunction. As a permanent colostomy is life altering, the patient should be referred to the enterostomal therapist for proper counseling and education. Equally as critical is the highest level of scrutiny of the preoperative imaging, specifically the MRI so that the most appropriate operation is offered to the patient. Additionally, anticipation of large perineal wounds, especially in those that undergo an extralevator excision should involve consultation with a plastic surgeon for proper reconstruction as described above. Lastly, frequent follow up and surveillance is the rule for these patients given the high level of morbidity of large perineal wounds and more importantly, local failure rates, respectively.

## Author Contributions

NG-H and DG led the overall organization and actual writing of the paper. JM was in charge of the final review and editing of the paper. All authors contributed to the article and approved the submitted version.

## Conflict of Interest

The authors declare that the research was conducted in the absence of any commercial or financial relationships that could be construed as a potential conflict of interest.

## References

[1] Campos FG Habr-Gama A Nahas SC Perez RO. Abdominoperineal excision: evolution of a centenary operation. Dis Colon Rectum. (2012) 55:844–53. 10.1097/DCR.0b013e31825ab0f7 22810469

[2] Hawkins AT Albutt K Wise PE Alavi K Sudan R Kaiser AM. Abdominoperineal resection for rectal cancer in the twenty-first century: indications, techniques, and outcomes. J Gastrointest Surg. (2018) 22:1477–87. 10.1007/s11605-018-3750-9 29663303

[3] Miles WE A method of performing abdomino-perineal excision for carcinoma of the rectum and of the terminal portion of the pelvic colon. Lancet. (1908) 2:1812–3. 10.1016/S0140-67360099076-7 5001853

[4] Miles WE. The radical abdomino-perineal operation for cancer of the pelvic colon. BMJ. (1910) 11:941–3. 19988156

[5] Schmitz RL Nelson PA Martin GB Boghossian HM. Synchronous (two-team) abdominoperineal resection of the rectum. AMA Arch Surg. (1958) 77:492–7. 10.1001/archsurg.1958.04370010024003 13582408

[6] Lloyd-Davies OV. Oswald Vaughan Lloyd-Davies 1905-1987. Lithotomy-Trendelenburg position for resection of rectum and lower pelvic colon. Dis Colon Rectum. (1989) 32:172–5. 10.1007/BF02553836 2644109

[7] Buckwalter JA. The case for the “two team” abdominoperineal resection. Am J Surg. (1962) 103:90–3. 10.1016/0002-9610(62)90021-1 13874333

[8] Lockhart-Mummery JP. Two hundred cases of cancer of the rectum treated by perineal excision. BJS. (1926) 14:110–24. 10.1002/bjs.18001453126365488

[9] Gray JH The relation of lymphatic vessels to the spread of cancer. BJS. (1939) 26:462–95. 10.1002/bjs.18002610303

[10] Grinnell RS. The lymphatic and venous spread of carcinoma of the rectum. Ann Surg. (1942) 116:200–16. 10.1097/00000658-194208000-00005 17858082 PMC1543804

[11] Mukkai Krishnamurty D Wise PE. Importance of surgical margins in rectal cancer. J Surg Oncol. (2016) 113:323–32. 10.1002/jso.24136 27094456

[12] Heald RJ. The 'Holy Plane' of rectal surgery. J R Soc Med. (1988) 81:503–8. 10.1177/014107688808100904 3184105 PMC1291757

[13] Heald RJ Moran BJ Ryall RD Sexton R MacFarlane JK. Rectal cancer: the basingstoke experience of total mesorectal excision, 1978-1997. Arch Surg. (1998) 133:894–9. 10.1001/archsurg.133.8.894 9711965

[14] McAnena OJ Heald RJ Lockhart-Mummery HE. Operative and functional results of total mesorectal excision with ultra-low anterior resection in the management of carcinoma of the lower one-third of the rectum. Surg Gynecol Obstet. (1990) 170:517–21. 1693016

[15] Quirke P Steele R Monson J Grieve R Khanna S Couture J. Effect of the plane of surgery achieved on local recurrence in patients with operable rectal cancer: a prospective study using data from the MRC CR07 and NCIC-CTG CO16 randomised clinical trial. Lancet. (2009) 373:821–8. 10.1016/S0140-6736(09)60485-2 19269520 PMC2668948

[16] Scott N Jackson P al-Jaberi T Dixon MF Quirke P Finan PJ. Total mesorectal excision and local recurrence: a study of tumour spread in the mesorectum distal to rectal cancer. Br J Surg. (1995) 82:1031–3. 10.1002/bjs.1800820808 7648142

[17] Nagtegaal ID Quirke P. What is the role for the circumferential margin in the modern treatment of rectal cancer? J Clin Oncol. (2008) 26:303–12. 10.1200/JCO.2007.12.7027 18182672

[18] Monson JR Probst CP Wexner SD Remzi FH Fleshman JW Garcia-Aguilar J. Failure of evidence-based cancer care in the United States: the association between rectal cancer treatment, cancer center volume, and geography. Ann Surg. (2014) 260:625–31. 10.1097/SLA.0000000000000928 25203879

[19] Dietz DW. Multidisciplinary management of rectal cancer: the OSTRICH. J Gastrointest Surg. (2013) 17:1863–8. 10.1007/s11605-013-2276-4 23884558

[20] Chessin DB Hartley J Cohen AM Mazumdar M Cordeiro P Disa J. Rectus flap reconstruction decreases perineal wound complications after pelvic chemoradiation and surgery: a cohort study. Ann Surg Oncol. (2005) 12:104–10. 10.1245/ASO.2005.03.100 15827789

[21] Danielsen AK Burcharth J Rosenberg J. Patient education has a positive effect in patients with a stoma: a systematic review. Colorectal Dis. (2013) 15:e276–83. 10.1111/codi.12197 23470040

[22] Kasparek MS Hassan I Cima RR Larson DR Gullerud RE Wolff BG. Quality of life after coloanal anastomosis and abdominoperineal resection for distal rectal cancers: sphincter preservation vs quality of life. Colorectal Dis. (2011) 13:872–7. 10.1111/j.1463-1318.2010.02347.x 20545966

[23] Camilleri-Brennan J Steele RJ. Quality of life after treatment for rectal cancer. Br J Surg. (1998) 85:1036–43. 10.1046/j.1365-2168.1998.00808.x 9717993

[24] Guren MG Eriksen MT Wiig JN Carlsen E Nesbakken A Sigurdsson HK Quality of life and functional outcome following anterior or abdominoperineal resection for rectal cancer. Eur J Surg Oncol. (2005) 31:735–42. 10.1016/j.ejso.2005.05.004 16180267

[25] Fleshman J Branda M Sargent DJ Boller AM George V Abbas M Effect of laparoscopic-assisted resection vs open resection of stage ii or iii rectal cancer on pathologic outcomes: the ACOSOG Z6051 randomized clinical trial. JAMA. (2015) 314:1346–55. 10.1001/jama.2015.10529 26441179 PMC5140087

[26] Fleshman J Branda ME Sargent DJ Boller AM George VV Abbas MA. Disease-free survival and local recurrence for laparoscopic resection compared with open resection of stage II to III rectal cancer: follow-up results of the ACOSOG Z6051 randomized controlled trial. Ann Surg. (2019) 269:589–95. 10.1097/SLA.0000000000003002 30080730 PMC6360134

[27] Stevenson AR Solomon MJ Lumley JW Hewett P Clouston AD Gebski VJ. Effect of laparoscopic-assisted resection vs open resection on pathological outcomes in rectal cancer: the ALaCaRT randomized clinical trial. JAMA. (2015) 314:1356–63. 10.1001/jama.2015.12009 26441180

[28] West NP Anderin C Smith KJ Holm T Quirke P. Multicentre experience with extralevator abdominoperineal excision for low rectal cancer. Br J Surg. (2010) 97:588–99. 10.1002/bjs.6916 20186891

[29] Han JG Wang ZJ Wei GH Gao ZG Yang Y Zhao BC. Randomized clinical trial of conventional versus cylindrical abdominoperineal resection for locally advanced lower rectal cancer. Am J Surg. (2012) 204:274–82. 10.1016/j.amjsurg.2012.05.001 22920402

[30] Prytz M Angenete E Bock D Haglind E. Extralevator abdominoperineal excision for low rectal cancer–extensive surgery to be used with discretion based on 3-year local recurrence results: a registry-based, observational national cohort study. Ann Surg. (2016) 263:516–21. 10.1097/SLA.0000000000001237 25906414 PMC4741394

[31] Han JG Wang ZJ Qian Q Dai Y Zhang ZQ Yang JS. A prospective multicenter clinical study of extralevator abdominoperineal resection for locally advanced low rectal cancer. Dis Colon Rectum. (2014) 57:1333–40. 10.1097/DCR.0000000000000235 25379997

[32] Bianco F Romano G Tsarkov P Stanojevic G Shroyer K Giuratrabocchetta S. Extralevator with vs nonextralevator abdominoperineal excision for rectal cancer: the RELAPe randomized controlled trial. Colorectal Dis. (2017) 19:148–57. 10.1111/codi.13436 27369739

[33] Carpelan A Karvonen J Varpe P Rantala A Kaljonen A Grönroos J. Extralevator versus standard abdominoperineal excision in locally advanced rectal cancer: a retrospective study with long-term follow-up. Int J Colorectal Dis. (2018) 33:375–81. 10.1007/s00384-018-2977-y 29445870

[34] Mesquita-Neto JWB Mouzaihem H Macedo FIB Heilbrun LK Weaver DW Kim S. Perioperative and oncological outcomes of abdominoperineal resection in the prone position vs the classic lithotomy position: a systematic review with meta-analysis. J Surg Oncol. (2019) 119:979–86. 10.1002/jso.25402 30729542 PMC6844252

[35] Han JG Wang ZJ Gao ZG Wei GH Yang Y Zhai ZW. Perineal wound complications after extralevator abdominoperineal excision for low rectal cancer. Dis Colon Rectum. (2019) 62:1477–1484. 10.1097/DCR.0000000000001495 31567926

[36] de Campos-Lobato LF Stocchi L Dietz DW Lavery IC Fazio VW Kalady MF Prone or lithotomy positioning during an abdominoperineal resection for rectal cancer results in comparable oncologic outcomes. Dis Colon Rectum. (2011) 54:939–46. 10.1097/DCR.0b013e318221eb64 21730781

[37] Blok RD de Jonge J de Koning MA van de Ven AWH van der Bilt JDW van Geloven AAW. Propensity score adjusted comparison of pelviperineal morbidity with and without omentoplasty following abdominoperineal resection for primary rectal cancer. Dis Colon Rectum. (2019) 62:952–9. 10.1097/DCR.0000000000001349 30747743

[38] Qi XY Cui M Liu MX Xu K Tan F Yao ZD. Extralevator abdominoperineal excision versus abdominoperineal excision for low rectal cancer: a meta-analysis. Chin Med J. (2019) 132:2446–56. 10.1097/CM9.0000000000000485 31651517 PMC6831059

[39] Lehtonen T Räsänen M Carpelan-Holmström M Lepistö A. Oncological outcomes before and after the extralevator abdominoperineal excision era in rectal cancer patients treated with abdominoperineal excision in a single centre, high volume unit. Colorectal Dis. (2019) 21:183–90. 10.1111/codi.14468 30411461

[40] Zhang Y Wang D Zhu L Wang B Ma X Shi B. Standard versus extralevator abdominoperineal excision and oncologic outcomes for patients with distal rectal cancer: a meta-analysis. Medicine. (2017) 96:e9150. 10.1097/MD.0000000000009150 29384902 PMC6393134

[41] Negoi I Hostiuc S Paun S Negoi RI Beuran M. Extralevator vs conventional abdominoperineal resection for rectal cancer-a systematic review and meta-analysis. Am J Surg. (2016) 212:511–26. 10.1016/j.amjsurg.2016.02.022 27317475

[42] Güven HE Aksel B. Is extralevator abdominoperineal resection necessary for low rectal carcinoma in the neoadjuvant chemoradiotherapy era? Acta Chir. (2019). 10.1080/00015458.2019.1634925. [Epub ahead of print]. 31250735

[43] De Nardi P Summo V Vignali A Capretti G. Standard versus extralevator abdominoperineal low rectal cancer excision outcomes: a systematic review and meta-analysis. Ann Surg Oncol. (2015) 22:2997–3006. 10.1245/s10434-015-4368-8 25605518

